# The Role of Extracellular Vesicles in Cancer–Nerve Crosstalk of the Peripheral Nervous System

**DOI:** 10.3390/cells11081294

**Published:** 2022-04-11

**Authors:** Yuanning Guo, Ziv Gil

**Affiliations:** 1 Rappaport Family Institute for Research in the Medical Sciences, Technion—Israel Institute of Technology, Haifa 31096, Israel; stamduck@126.com; 2 Head and Neck Institute, The Holy Family Hospital Nazareth, Nazareth 1641100, Israel

**Keywords:** exosome, microvesicle, cancer–nerve crosstalk, cancer neuroscience, neural influences in cancer (NIC), axonogenesis, neurogenesis, innervation, tumor microenvironment (TME), peripheral nervous system (PNS)

## Abstract

Although the pathogenic operations of cancer–nerve crosstalk (e.g., neuritogenesis, neoneurogensis, and perineural invasion—PNI) in the peripheral nervous system (PNS) during tumorigenesis, as well as the progression of all cancer types is continuing to emerge as an area of unique scientific interest and study, extensive, wide-ranging, and multidisciplinary investigations still remain fragmented and unsystematic. This is especially so in regard to the roles played by extracellular vesicles (EVs), which are lipid bilayer-enclosed nano- to microsized particles that carry multiple-function molecular cargos, facilitate intercellular communication in diverse processes. Accordingly, the biological significance of EVs has been greatly elevated in recent years, as there is strong evidence that they could contribute to important and possibly groundbreaking diagnostic and therapeutic innovations. This can be achieved and the pace of discoveries accelerated through cross-pollination from existing knowledge and studies regarding nervous system physiology and pathology, as well as thoroughgoing collaborations between oncologists, neurobiologists, pathologists, clinicians, and researchers. This article offers an overview of current and recent past investigations on the roles of EVs in cancer–nerve crosstalk, as well as in neural development, physiology, inflammation, injury, and regeneration in the PNS. By highlighting the mechanisms involved in physiological and noncancerous pathological cellular crosstalk, we provide hints that may inspire additional translational studies on cancer–nerve interplay.

## 1. Introduction

Naturally released by cells, extracellular vesicles (EVs), are membranous nano- to microsized structures enclosed by a lipid bilayer, which carry molecular cargos. EVs are heterogeneous due to their large variation in size (from 30 to 10,000 nm in diameter) and the biogenesis pathways from which they arise, as well as their cellular origins, cargos, and functions [1]. The differences in size, synthesis route, and cargos allow EVs to be principally divided as exosomes (30 to 160 nm, endosomal origin), microvesicles (50 nm to 1 μm, plasma membrane origin), and apoptotic bodies [1,2,3]. With this said, EV research is a very new and fast-developing area of investigation. Accordingly, due to differences in and the limitations of EV isolation methods, the overlap of EV subtypes in terms of size, and the as-yet unresolved confusion regarding EV nomenclature, for the sake of simplicity and clarity, in this review we will use the term EV as a catchall to describe the general concept of all EV subtypes.

Physiological and pathological intercellular communication through EVs is extremely common and, therefore, significant [4,5], particularly because isolated EVs offer protection to vulnerable cargos, which allows these cargos to remain stable in complex local microenvironments and during transportation across long distances. For these reasons, among others, the employment, as well as the targeting of EVs or EV-driven intercellular communication has gradually been exhibiting potential diagnostic and therapeutic promise [1,2].

It has been established during the past two decades that molecular intercellular crosstalk via the release, transference, and internalization of EVs containing multiple types of molecular cargo is a novel mechanism, which is distinct from physical contact, as well as classical intercellular crosstalk methods (i.e., endocrine, paracrine, and autocrine). The molecular cargos of EVs can be extremely diverse, ranging from proteins, nucleic acids (especially noncoding RNAs), lipids, and metabolites to organelles from parent cells. Cargos carried by EVs are not randomly selected. Rather, cargo choice is dependent upon the type and status of parent cells, as well as how those cells respond to microenvironmental alterations. Consequently, cargo differences can induce a variety of outcomes within recipient cells and local environments [1,2,3].

Accumulating evidence has been demonstrating that there is a multiplicity of pathogenic pathways that facilitate intercellular communication, systematically or locally within the tumor microenvironment (TME), between cancer and the nervous system. This burgeoning field of cancer neuroscience or onconeurology has attracted increasing attention and is encouraging multidisciplinary collaborative efforts involving both cancer biologists and clinical oncologists [6,7,8]. The elaborate crosstalk between neurons, nerve fibers, glial cells, cancer cells, stroma, and immune cells contributes to almost all tumor progression phases and related neural symptoms: initial, proliferation, invasion, metastasis, and cancer-associated pain [9,10]. Cancer–nerve crosstalk can take place in the PNS. It can also occur within the central nervous system (CNS), where pathogenesis is more complicated. For this reason, neither the CNS nor the role of cancer–nerve crosstalk within it will be addressed in this article. Pathogenic processes can occur either by direct crosstalk between nerves and cancer cells, or via indirect means involving other cellular components, such as glia, stroma, or immune cells [11]. As regards cancer–nerve crosstalk in the peripheral nervous system (PNS), these can be broken down into three categories [9,10]:

- Perineural invasion (PNI), a process by which tumor cells actively invade a mature nerve and present within either its endoneurial, perineurial, or epineurial membrane layers [12,13,14].
- Neuritogenesis, axonogenesis, and innervation all describe the process by which newly formed axons (or neurites) actively project themselves onto cancer cells and thereby support cancer progression via multiple neurotransmitters or neuropeptides [9,15,16]. Neuritogenesis is the initial and foremost event involved in neuronal morphogenesis before transforming into both axonogenesis and dendritogenesis. Nevertheless, in all of the literature we reviewed on cancer–nerve crosstalk, as well as that of nerve injury and regeneration, it appears as if the term neuritogenesis is frequently being used to convey a broader concept. In other words, whether in vitro or in vivo, when inducing the neurite outgrowth of neurons or DRGs, researchers may actually be observing dendritogenesis, axonogenesis, or the narrower definition of neuritogenesis, but will simply use the latter term as a catchall. For the purposes of this article, we shall do the same. With this understood, as regards the more narrow-focused definition of neuritogenesis, it is important to understand that dendritogenesis is referring to the outgrowth of dendrites and which, to date, no connection to cancer–nerve crosstalk has been found. Axonogenesis emphasizes the outgrowth of axons [17], which is more specifically descriptive of the actual process of in vivo cancer–nerve crosstalk (as well as that of nerve injury and regeneration) that results from newly formed neural twigs (axons) sprouting toward target cells. The concept of innervation focuses more on nerve-derived functions, where their influence on target (innervated) cells depends on different types of nerves and target cells, as well as the several neurotransmitters or neuropeptides that are released [9,10,16]. As such, when necessary, to emphasize neural functionality, we will use the term innervation.
- Neurogenesis or neoneurogenesis sometimes convey broad meanings, which refer not only to the new formation of neurons, but also to neuritogenesis or axonogenesis. Accordingly, the exact meaning of these concepts is context-dependent [10,18,19]. Nevertheless, in cancer–nerve crosstalk literature, neoneurogenesis best describes the increasing number of neurons in the TME from neural progenitors [18] and is the term we shall use in this review.

Although there have been numerous published cancer–nerve crosstalk studies in recent years, they are fragmented and unsystematic. Therefore, it is urgent for more neurobiologists to participate in cancer neuroscience and, through their cooperative efforts with oncologists, a more comprehensive and complete knowledge of this field will emerge. An important way to accelerate development in this area is to seek clues about and investigate the potential mechanisms of cancer–nerve crosstalk through cross-pollination from existing knowledge and studies regarding nervous system physiology and pathology. This will likely bear fruit as the biological behaviors of cancer always mimic and hijack physiological processes or components thereof, especially during development and regeneration [11]. The similarities and parallel relationship between cancer–nerve crosstalk and neuroscience can inspire us toward further research into potential diagnostic and therapeutic strategies both for cancer and neural diseases.

In this article, we will offer a digest of what the literature regarding EV functions in cancer–nerve crosstalk has already revealed, although there are only four published or preprint original studies available [20,21,22,23]. Thereafter, we will systematically review EV-related studies on neurobiological processes, from which we will provide hints to inspire future translational studies about the roles played by EVs—from the nervous system to cancer–nerve crosstalk in pathogenesis, diagnostic applications, and therapeutics.

## 2. Cancer–Nerve Crosstalk and Its Relationships with Neural Physiology, Inflammation, Injury, and Regeneration

**Figure 1:**
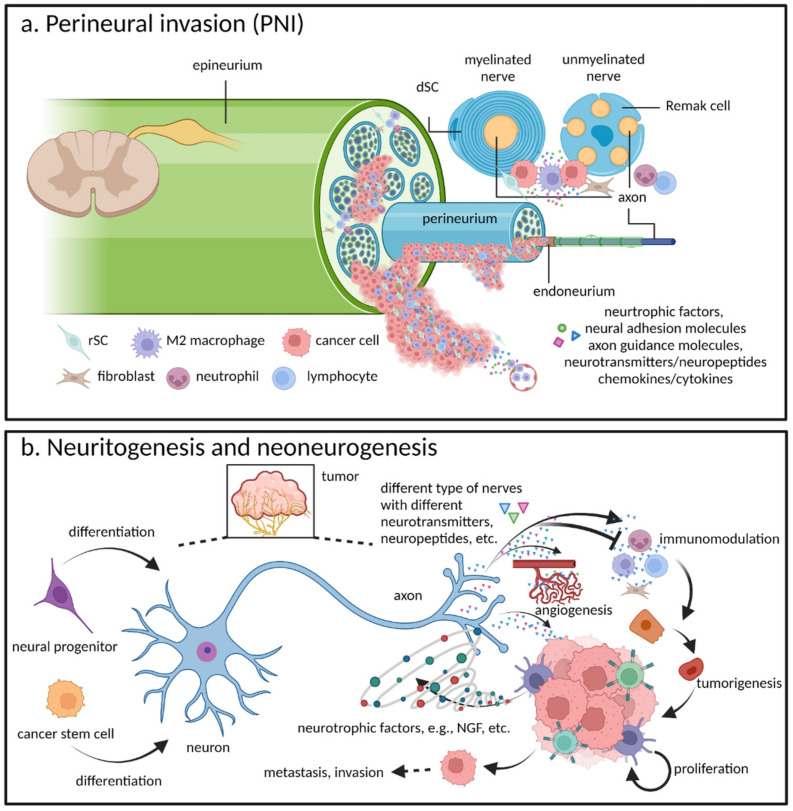
An illustration of the multiple means of pathogenesis involved in cancer–nerve crosstalk: (**a**) Macrophages, Schwann cells, fibroblasts, immunocytes, and other stroma cells have been reported to be involved in perineural invasion (PNI), where multiple neurotropic, neuroadhesion, axon guidance factors, neurotransmitters/neuropeptides, and cytokines/chemokines participate in cellular interactions; (**b**) Induced and promoted by cancer cells, neuritogenesis promotes tumorigenesis and cancer progression by directly activating cancer cells or inducing angiogenesis, modulating the immunomicroenviornment, and so forth through means of multiple neurotransmitters or neuropeptides. Neoneurogenesis occurs when neural progenitors (or, in some instances, cancer stem cells) differentiate to mature neurons and is followed by tumor-induced neuritogenesis. rSC: repair Schwann cell (or dedifferentiated Schwann cell); dSC: differentiated Schwann cell; NGF: neural growth factor.

The crosstalk between cancers and nerves can be achieved by direct interactions between cancer cells and axons/neurons (direct model). However, in other situations, indirect crosstalk can take place through the participation of glia cells, immunocytes, endothelial cells, or other stroma cells, thus creating a comprehensive and suitable microenvironment to attract nerves or cancer cells (indirect model) [9,11] (Figure 1).

Moreover, a comprehensive understanding of the literature on neurobiology strongly suggests that practically all cancer–nerve crosstalk mechanisms can be mapped to neural biological processes. Accordingly, as neuroscience is still a fast-growing area of research, it should be considered of great significance and a necessity for cancer neuroscience researchers to pursue this mapping with great vigor. In this regard, one hurdle to overcome is the fact that up until quite recently, oncology has tended to operate within its own, isolated spheres of research, interacting very little with the wider field of neuroscience.

### 2.1. Perineural Invasion: A Critical Pathway for Tumor Spread

In the mid-1800s, pathological anatomists first discovered the phenomenon of PNI, which is now widely accepted as a distinct entity alongside lymphovascular invasion, where PNI is defined as the act of tumor cells invading and spreading along nerves within the epineurial, perineurial, or endoneurial spaces of the neural sheath [12,13,24] (Figure 1a). PNI involvement has been associated with poor outcomes and low rates of survival in relation to a large spectrum of malignant tumors, including many kinds of epithelial-originating solid tumors from a variety of organs (keratinocyte carcinomas, adenocarcinomas, and basal cell carcinomas), melanomas, and even some types of leukemia, lymphoma, and sarcoma [13,24,25,26,27,28,29,30,31,32,33]. Different PNI incidence rates exist for different tumors. The highest rate is for pancreatic cancer (80–100%), where PNI involvement in this cancer type is, to date, the most well studied [34,35,36].

Within the past three decades, researchers have become increasingly interested in understanding the molecular mechanisms related to cellular crosstalk in the perineural niche, which drives tumorigenesis, invasion, and metastasis [12]. An outdated theory once regarded PNI as an extension of cancer lymphatic spread/metastasis, while an equally obsolete theory regarded PNI as the “path of low resistance.” Presently, PNI is acknowledged as the active invasion of cancer cells into nerves via cellular crosstalk instead of simple diffusion, while discoveries related to the active roles played by stroma cells have launched a new era of PNI research. Cancer–nerve crosstalk investigations, especially those which have examined inflammatory reactions and neurotrophic assistance promoted during PNI by macrophages and Schwann cells (SCs, which are the main peripheral glia cells) imply that cancer invasion into nerves mimics the process of nerve injury and regeneration [34,37,38] (Figure 1a).

The crosstalk between cancer cells and nerves (including neurons, axons, SCs, immunocytes, and other stroma cells)—through neurotrophins, glial cell-derived neurotrophic factor (GDNF), chemokines/cytokines, midkine family molecules, neural adhesion molecules, axon guidance molecules, neurotransmitters/neuropeptides, and so forth —mimics the interactions by activated SCs, macrophages, injured axons, or target (innervated) tissues and nerves [14,39,40,41,42,43,44,45,46,47].

Our group, along with collaborative partners, have been pioneers in this field and have discovered a series of relatively complete cellular and molecular crosstalk mechanisms [14,34,48,49,50,51,52,53,54,55]. Among these, we found that a prominent component of the perineural microenvironment, macrophages, are (1) recruited by PNI niche-released chemokines and (2) polarized to tumor-associated phenotype (M2) (Figure 1a). In this process, CSF-1 secretion by cancer cells mediates macrophage chemotaxis into the neural niche by activation of CSF-1 receptor (CSF-1R) [52]. These macrophages are actively involved in mediating PNI via the secretion of the GDNF, which attracts and activates cancer cell migration toward nerves by binding with the GDNF receptors, RET/GFRα1. Soluble GFRα1 is released by nerves (possibly via neurons, axons, SCs, fibroblasts, or endoneurial macrophages) and cooperates with macrophage-secreted GDNF to enhance RET activation and PNI [53]. Targeting the RET/Ras/ERK pathway can suppress nerve invasion and may contribute to better outcomes in pancreatic cancer [50].

Apart from macrophages, SCs have also been found to contribute to PNI. The critical, active roles played by SCs in PNI and cancer have been confirmed by several independent studies. All have pointed out the important fact that SCs in the TME present a dedifferentiated or transdifferentiated phenotype, which is probably produced by the alteration of PNI-induced neural environmental signals or damage to/displacement of nerves during tumor expansion and invasion (Figure 1a). Before cancer invades nerves (or even during the intraepithelial neoplastic stage), SCs can actively migrate toward cancer cells. This interaction seems to be predominantly orchestrated by the chemoattractive action of cancer-releasing nerve growth factor (NGF) upon SC p75NTR [37]. Additionally, SCs can foster PNI by secreting L1 cell adhesion molecule (L1CAM), which not only attracts cancer cells through the activation of MAPK signaling, but also upregulates metalloproteinase by STAT3 activation [55]. Apart from paracrine signals, upon direct contact, SCs have been found to induce the formation of cancer cell protrusions in their direction and intercalate between cancer cells, leading to cancer cell dispersion. The initiation of these processes was largely contributed to by SC expression of neural cell adhesion molecule 1 (NCAM1), which ultimately promoted PNI [34]. Moreover, SCs secrete CCL2 and recruit inflammatory monocytes/macrophages from the circulation system via CCR2, which also facilitates tumor invasion into and along the nerve through a cathepsin B-mediated disruption of the protective perineurium [51,54]. In cutaneous melanoma, although PNI is not crucial for tumor progression, nor significant to patient outcomes, SCs can still transform into a dedifferentiated, active state, which is likely due to the destruction or displacement of existing cutaneous sensory nerves. These SCs are functionally similar to repair SCs (rSCs) that participate in the process of Wallerian degeneration (discussed further below), as they present characteristics such as enhanced motility, ECM reorganization, neurotrophic factor release, and macrophage attraction/M2 polarization [56]. SCs activated in the TME can mimic a reactive gliosis status, by which SCs engage in the interplay between spinal astrocyte and microglia, and contribute to analgesia, thus obfuscating cancer progression [38].

These PNI-promoting macrophage and SC reactions imitate many vital pathological processes of peripheral nerve injury and regeneration [40,46,47,57]. Indeed, PNI is a special nerve injury where nerves are damaged by tumor invasion [14]. Physiologically, SCs and macrophages are the main non-neuronal cells in nerves [46]. Unlike the central nervous system (CNS), the relatively easily damaged PNS has remarkable post-injury, self-reparative abilities, which largely rely on non-neuronal cells, especially SCs and macrophages. Nerve injuries usually result from compression, crushing, trauma, laceration, chemical irritation, and stretching. According to Seddon’s classification system of peripheral nerve injuries, there are three grades of nerve injury: neuropraxia, axonotmesis, and neurotmesis. The latter two are more serious and common, and are usually caused by crushing and transection, respectively, which progressively undergo Wallerian degeneration [58,59], where SC and macrophages are completely activated [41]. This may explain the reason why the invasion of cancer cells into the endoneurium is associated with a worse prognosis than invasion around or within other nerve layers [47]. 

SCs are first responders to peripheral nerve injury. Soon after such injuries, SCs in distal-detached axons begin to dedifferentiate, a process whereby SCs halt the production of myelin proteins, upregulate dedifferentiated and regeneration-associated genes (e.g., GFAP, NCAM1, L1CAM, neurotrophic factors, and their receptors, such as p75NTR), and begin to proliferate, elongate and branch, thus forming regenerative tracks (Büngner bands) [40,41,43]. These activated SCs are known as repair SCs (rSCs), as they exhibit a dedifferentiated, non-myelinated, neurotrophic, and proliferative phenotype [39,40,43,45], which is similar to that which is observed during PNI. rSCs also stimulate endoneurial-resident macrophages and actively attract circulating macrophages through the secretion of multiple chemokines and interleukins, including CCL2, TNF-α, IL-6, and LIF. These activated macrophages, in turn, further stimulate SC proliferation and other neural repair functions. As a result, SCs collaborate with macrophages to clear myelin debris in the early stages of nerve injury and regeneration, and together amplify chemoattractive and cytokine signals to recruit and activate increasing amounts of stroma and immune cells into the nerve injury niche. In later stages, macrophages are educated and polarize to an immunosuppressive phenotype (M2), which establishes and maintains immunobalance with the help of other immunocytes (i.e., Th1 and Th2), and cooperates with rSCs to promote axon regeneration and reinnervation [40,41,46,57].

Other cells, such as fibroblasts and mast cells, which have been reported to be associated with and as facilitating PNI, are also resident in the nerve niche, playing critical roles in nerve physiology, as well as nerve injury and regenerative processes [42,46,60]. With greater understanding about nerve structures and histology, it becomes increasingly possible to determine the types of microenvironments and cells that cancer cells will encounter when they invade into different layers, or at different locations of nerve bundles (at ganglions, distal nerve fibers, or nerve terminals) [47]. Therefore, increasing knowledge about nerve injury and regeneration helps to establish new paradigms and points of reference toward the further study of PNI.

### 2.2. Neuritogenesis and Neoneurogenesis: Additional Components of Cancer–Nerve Crosstalk Pathogenesis

Apart from angiogenesis and lymphangiogenesis, evidence indicates that neuritogenesis and neoneurogenesis also contribute largely to tumorigenesis and cancer progression [10] (Figure 1b). Neoneurogenesis, which describes the increasing amounts of neurons in the TME by neural progenitors, has been reported in prostate cancer [18] and may be involved in those tumors in which neural progenitors are abundant and their renewal/proliferation is common (e.g., enteric nervous system in the digestive tract) [61,62] (Figure 1b). Additionally, unique cases revealed by Lu et al. demonstrated that cancer stem cells from gastric, colorectal, and lung adenocarcinomas were able to differentiate into sympathetic- or parasympathetic-like neurons, thus further promoting cancer progression due to their intercellular interplay with cancer cells [63] (Figure 1b). Despite the fact that there exists the possibility that neoneurogenesis-produced neuron somas can be directly involved in intercellular interactions, studies of neoneurogenesis in cancer have demonstrated that the cancer-promotion effect triggered by newly formed nerves is mainly due to cancer neuritogenesis and innervation following neoneurogenesis [18,63] (Figure 1b). Within the past ten years, attention has been focused on the roles of neuritogenesis in and neural regulation of local organ microenvironments as they relate to immunity and cancer pathogenesis [7,64]. Similar to the different roles that have been reported about autonomic, sensory, and even motor nerves in organogenesis, tissue homeostasis, plasticity, and regeneration [65], the nervous system can also control malignant tumor initiation, growth, spread, recurrence, and therapeutic resistance [66].

In the early stages of tumorigenesis, neuritogenesis and neoneurogenesis are already present in preneoplastic lesions and contribute to the initiation of cancer. A 2019 study has suggested that neural progenitor cells are transported from the subventricular zone of the CNS into the circulatory system during cancer development. After reaching and infiltrating preneoplastic, primary, or metastatic tumor sites, these progenitors can further differentiate into new adrenergic neurons that promote carcinogenesis and metastasis [18] (Figure 1b). In addition, pancreatic adrenergic and nociceptive/sensory innervation, cholinergic nerves under gastric epithelia, as well as adrenergic nerves in the prostate, were found to increase dramatically when only intraepithelial neoplasia was apparent [67,68,69,70,71]. The neuritogenesis and innervation are partly contributed to by neurotrophic factors released by preneoplastic cells, and can directly activate cancer pathways or indirectly modulate activities within the TME (e.g., angiogenesis), which can further form a neurotransmitter-neurotrophin feedforward loop that promotes carcinogenesis. These protumorigenic interactions and signals, depend on different type of tumors, can be kept, enhanced, or adjusted in later cancer stages [9,15,69,70,71]. Besides regulating cancer proliferation, invasion, metastasis, angiogenesis, some neurotransmitters/neuropeptides can also be involved in immunoevasion, by inhibiting immunocytes via neurotransmitter/neuropeptide receptors [9] (Figure 1b).

Similar to PNI, cancer–nerve crosstalk in the context of neuritogenesis and neoneurogenesis not only occurs through direct communication (i.e., cancer-induced neuritogenesis via the release of NGF [70,71]), but also takes place indirectly via the neural regulation of other cells within the TME (e.g., immune and endothelial cells) [11,69,72] (Figure 1b).

The cellular interactions between cancer and various nerve types and organ microenvironments, and their effect upon cancer progression stages are multifaceted and complicated because the specific mechanisms are reliant upon any number of neurotransmitters, neural growth factors, and signaling cascades, as well as paracrine signaling and direct electrochemical communication [7,69] (Figure 1b). For instance, in two studies that used the same mouse models (KC and KPC), cholinergic signaling can directly and indirectly suppress pancreatic tumorigenesis and cancer stemness [73], while adrenergic nerves foster pancreatic cancer progression [71]. On occasion, a certain nerve type can both promote and inhibit cancer progression in a tumor type-dependent manner. In particular, the parasympathetic nervous system strongly promotes adenocarcinoma of the stomach (MNU-induced and INS-GAS mouse gastric models) and the prostate (xenograft and Hi-Myc mice) [69,70,74], while it inhibits the growth and progression of pancreatic adenocarcinoma [73].

Apart from the fact that different types of nerves and neurotransmitters can influence tumors in a variety of ways, the function of distinct nerve types that innervate cancer tissue is determined by its original anatomical physiology and specific microenvironment [7,75]. Here are three additional examples (Figure 1b): (1) Nerve terminals that directly innervate target cells not only support their physiological functions, but also drive their survival, proliferation, and development, as well as maintain the stem cell niche. Similar behaviors are also in evidence when cancer cells hijack nerve functions; (2) The nervous system directly regulates systemic and local immunocytes, as well as immunoinflammatory functions [64,76,77]. Accordingly, nerves can also regulate cancer progression via the immune system and local oncoimmunobiology [78,79]; and (3) neuritogenesis or innervation, or even neoneurogenesis in cancer offers evidence of wound-healing, neural regeneration, and reinnervation processes after tissue or nerve injury [18,41,44,80]. Namely, in nerve repair, neurotrophic and neural guidance signals and pathways among axons, SCs, immunocytes, and target cells also exist within cancer–nerve crosstalk [9,18,47]. As we have mentioned that PNI mimics the mechanisms involved in nerve injury and regeneration, we should emphasize that PNI, neuritogenesis, and neoneurogenesis are not completely independent processes because they often coexist [12,42]. As such, in cancer, similar to that which can be observed in neural repair biology, nerve injury, regeneration, and reinnervation are highly connected [41,44]. Indeed, neuritogenesis and PNI frequently occur in highly innervated organs [47] and display similar neurosignaling patterns [42,47].

Mapping of cancer–nerve crosstalk within the biology of the central, peripheral, and enteric nervous systems is warranted urgently, as revelations regarding their as-yet poorly characterized, complex anatomies can serve as crucial points of reference and guidance as regards the interplay of cancer nerves with EVs.

## 3. The Role of Extracellular Vesicles in Cancer–Nerve Crosstalk in the Peripheral Nervous System

**Figure 2:**
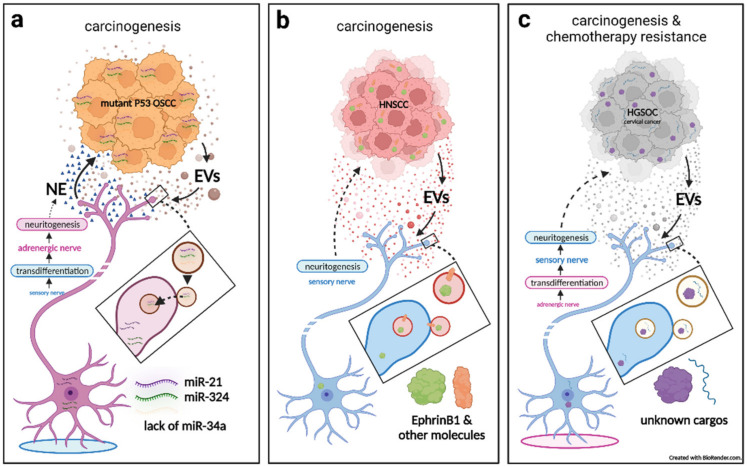
The three roles of extracellular vesicles (EVs) in cancer–nerve crosstalk are: (**a**) cancerous EV-delivered miRNAs (a combination of increasing miR-21, miR-324, and a lack of miR-34a) induce nerve reprogramming and neuritogenesis, which facilitates tumorigenesis; (**b**) cancerous EV-delivered axon guidance proteins (EphrinB1) promote sensory nerve neuritogenesis, which facilitates tumorigenesis; and (**c**) sensory neuritogenesis promotion by cancer EVs facilitates tumorigenesis, tumor-associated pain, and chemotherapeutic resistance. OSCC: oral squamous cell carcinoma: EV: extracellular vesicle; NE: noradrenaline; HNSCC: head and neck squamous cell carcinoma; HGSOC: high-grade serous ovarian carcinoma.

The research on EVs in cancer has been progressing more rapidly than have investigations into the relation of EVs to all other diseases [2]. EV transfer of molecules has obvious advantages as EVs not only protect their cargos from harsh microenvironments and serum, but also allow for their distant transportation [4,5]. Particularly, EVs can remain stable in serum, while low pH conditions in the TME typically facilitate the release and uptake of EVs [5,81]. Accordingly, it is unsurprising that EV functions have been found to bear a close association with many of the key hallmarks of cancer [82], especially as they relate to the TME [2,3,83,84,85,86]. Accumulating evidence suggests that the neural regulation (or nerve dependence) of cancer in the TME is becoming a new hallmark of the disease [6,7,8]. Besides the fact that EV transfer is favored in the TME, EV-based communication among cells in the neural microenvironment is also abundant since cell–cell transfer of molecules is necessary because neuronal soma cannot supply and support the constant demands of an entire neuron, particularly long-distance transportation to the axon [87,88]. Researchers have already found EVs playing some key roles in cancer–nerve interactions and communication [5,16,89,90] (Figure 2).

### 3.1. Cancerous Extracellular Vesicle-Delivered miRNAs Induce Reprogramming and Neuritogenesis of Nerves, Which in Turn Facilitates Tumorigenesis

Amit et al. published a notable study, which revealed that cancerous EV-delivered miRNA alterations induce the reprogramming, transdifferentiation, and neuritogenesis of sensory nerves, which then facilitates tumorigenesis [23,91] (Figure 2a). Specifically, they conducted a clinical analysis of oral squamous cell carcinoma (OSCC) samples and found that mutant p53 was associated with high adrenergic (sympathetic) nerve density, which was associated with poor clinical survival. The same association of p53 and adrenergic nerve density also existed in p53 knockout/mutation mouse OSCC models. They further discovered that the promotion of neurite growth was mainly provoked by p53 mutant OSCC-derived EVs, as they cocultured EV-containing medium of OSCC with trigeminal ganglion (TG) neurons and dorsal root ganglions (DRGs). However, EV-depleted or wild-type p53-conditioned medium did not induce neuritogenesis.

When injecting EVs from p53-deprived OSCC into wild-type mouse tumors, the researchers found that they grew larger with more adrenergic innervation, indicating that p53 status-altered EVs drive adrenergic neuritogenesis and tumorigenesis. Furthermore, there were no significant differences in the number and size of EVs from OSCC cells, whether mutant- or wild-type p53-derived, although their cargo miRNAs differed. Only the EVs secreted from mutant p53 tumors were lacking in miR-34a, which mainly contributed to neuritogenesis.

Next, they found that tumor growth can be inhibited by sensory denervation or pharmacological blockade of adrenergic receptors. However, in contrast to previous findings on prostate cancer mouse models [69], destroying adrenergic nerves before tumor inoculation neither inhibited adrenergic neuritogenesis nor blocked OSCC growth, but ablation of sensory nerves prevented these outcomes. This suggests that adrenergic neuritogenesis does not derive from already extant adrenergic neurons, but stem from neural reprogramming, transdifferentiation, and neuritogenesis from sensory nerves. Consequently, this process is found to be driven by the failed transfer of miR-34a but accompanied by the transferring of other neuritogenic miRNAs (miR-21 and miR-324), which together promote new neural networks in the TME and provide cancer progression signals (noradrenaline) [16,23,91].

### 3.2. Cancerous Extracellular Vesicle-Delivered Axon Guidance Proteins Promote Neuritogenesis of Sensory Nerves, Which in Turn Facilitates Tumorigenesis

Investigations of clinical and mouse head and neck squamous cell carcinoma (HNSCC, including OSCC) models led by Paola D. Vermeer revealed significantly higher innervation in cancer as compared with normal tissue [4,22]. However, neurogenically formed nerve twigs were sensory nerves (β-III tubulin+/TRPV1+), as opposed to adrenergic nerves. By using PC12 cells from a rat pheochromocytoma cell line in vitro, it was shown that PC12 neurite outgrowth can be highly induced by EVs both from patient tumors and serum. The use of mEERL mouse model-derived cancer or serum EVs can produce the same effects on PC12 cells. This mEERL model was created by injecting mEERL cells—which were derived from a mouse oropharyngeal epithelial cell line that stably expresses HPV16 viral E6 and E7 oncogenes, H-Ras and luciferase—into the hind limbs of the mice. However, blockade of EV release significantly attenuated both tumor innervation and growth [22]. When mEERL tumors were implanted in a transgenic mouse model that lacks TRPV1+ sensory nerves [20], tumor growth was significantly attenuated, which confirms that tumorigenesis is sensory nerve neuritogenesis-dependent [4].

Further exploration showed that neuritogenesis can be highly potentiated by an EV-packaged axonal guidance molecule, EphrinB1, which can engage Eph receptors on nerves and initiate neuritogenic signaling (Figure 2b). However, EphrinB1 was neither necessary nor sufficient for those EVs to induce neuritogenesis since blocking or genomic deletion of EphrinB1 did not significantly inhibit neurite outgrowth activity. Interestingly, human papillomavirus HPV (E6+) positive HNSCC-derived EVs may often express higher EphrinB1 levels and exhibit higher neurite outgrowth activity than HPV-negative HNSCC (although HPV infection is not a prerequisite for EV-induced neuritogenesis). The Vermeer team also offered in vitro evidence that EVs from other cancer-type cell lines (melanoma, colorectal and breast carcinomas) also harbor neuritogenic activity suggesting that tumor innervation, as induced by cancerous EVs, may be a common phenomenon [4,22].

### 3.3. Sensory Neuritogenesis Promotion by Cancer Extracellular Vesicles Facilitates Tumorigenesis, Tumor-Associated Pain, and Chemotherapy Resistance

The Vermeer team also conducted two other studies on EVs in cancer–nerve crosstalk from cervical [21] and from ovarian carcinomas [20] (Figure 2c). In the first of these, they explored issues related to TRPV1+ sensory nerves, whose density increases in association with carcinogenesis in human cervical tumors. Furthermore, the majority of cervical cancer patients suffer from significant pain [92], and increased TRPV1 expression in cervical carcinoma is consistent with patient pain data. Then, these investigators observed that EVs derived from cervical cancer cell lines can effectively stimulate neuritogenesis of PC12 cells. Meanwhile, EVs derived from HPV+ cell lines promote neurite outgrowth in excess of that which is produced from HPV− cell lines, which suggests that the phenomenon of cervical cancer-derived, EV-mediated neuritogenesis may be partially caused by the integration of HPV. In a large percentage of cervical cancer patients, HPV DNA integration into the host genome is observed [93,94], which results in worse clinical outcomes [94,95]. The authors suggested that the integration of HPV DNA is associated with the disruption of both viral oncogene expression and the host genome, which may alter the expression of oncogenes or tumor suppressor genes [96], thus leading to an alteration of cargos and/or the function(s) of EVs. This implication has been supported by silencing the HPV viral oncogenes, E6 and E7, in HeLa cells [97,98]. Combined with HPV’s association with HNSCC EV-inducing neuritogenesis [22], the role of virus infection, especially HPV integration in altering EV-mediated neurite outgrowth, merits further exploration (e.g., cargo alteration and related mechanisms).

In another study, it was reported that high-grade serous ovarian carcinomas (HGSOCs) are also sensory innervated by an EV-mediated process that contributes to carcinogenesis and resistance to chemotherapy [20]. They found HGSOC is highly innervated by TRPV1+ sensory nerves instead of sympathetic nerves, which is in opposition to that which is typically found in normal fallopian tubes and ovaries (i.e., sympathetic+ and sensory−). This phenomenon also presented on their syngeneic mouse model of HGSOC. Functional neuronal connections were detected in both human and mouse HGSOCs as tumor tissue evidence electrical activity upon electrophysiological stimulation. Thus, sensory neuritogenesis on HGSOCs occurs as a result of carcinogenesis rather than by default native nerves, with newly formed neural circuits displaying functional electrophysiological features. This fact is similar to the results reported by Amit et al. on the transdifferentiation phenomenon from sensory innervation to a sympathetic innervation during OSCC development [23,91].

Then, it was found that the neuritogenic effect is driven by cancer-specific, EV-derived molecules. The researchers proved this by extracting and purifying EVs from the conditioned media of an isogenic set of cell lines: FT33-Tag (an immortal normal human fallopian tube secretory cell line) and two transformed tumorigenic cell lines (FT33-Myc and FT33-Ras), which stably express Myc and Ras oncogenes, respectively. Importantly, EVs from FT33-Myc and FT33-Ras cells induced robust neuritogenesis of PC12 cells while FT33-Tag EVs could not. They also verified that robust neuritogenic effects could be produced by employing more routinely used HGSOC cell lines, as compared to normal fallopian tube cell lines. Furthermore, by comparing a regular HGSOC model with a TRPV1+ sensory nerve-deprived transgenic model, they suggested that tumor-infiltrating TRPV1 sensory nerves contribute to tumor growth, particularly carboplatin resistance, which thus results in worse survival. By reviewing and analyzing clinical data, they found neoadjuvant-treated residual lesion samples had an increased presence of sensory twigs, suggesting that chemotherapy may promote sensory neuritogenesis. Accordingly, the problem—when a recurrent disease goes from being initially sensitive to treatment to treatment resistant—may be due to the gradual inducement of sensory neuritogenesis [20].

The researchers then extracted EVs from another set of isogenic cell lines, which consisted of a parental platinum-sensitive cell line and two, evolved platinum-resistant cell lines. Consequently, EVs derived from one of the resistant lines (established via intermittent exposure to increasing doses of cisplatin) induced significantly more neuritogenesis from PC12 cells than EVs derived from the sensitive parental line or the other resistant line (established via continuous cisplatin exposure). These facts suggest that the stress caused by intermittent chemotherapy pushes tumor cells to evolve a higher capacity to induce sensory innervation by altering EV cargos, which results in tumorigenesis, treatment resistance, and poor outcomes [20].

It is probable that due to the intense impact of sensory nerve-dominant innervation in cervical and ovarian carcinoma, studies have found that in the late stages of both cancer types, pain is one of the most prominent symptoms from which patients grievously suffer [20,21]. Accordingly, cancer-inducing nociception/sensory neuritogenesis is also a lengthy process that eventually causes pain to emerge as a principal symptom. Further study is required to determine if the same holds true for pancreatic cancer, HNSCC, and other pain-intensive or nociceptive/sensory-dominant cancers [22].

Some cancer types (e.g., prostate, breast, and stomach) induce sympathetic and parasympathetic neuritogenesis, and therefore this issue should be investigated further. Accordingly, in the study of the role of EVs in cancer–nerve crosstalk, it is important to characterize the different types and behaviors of nerve innervation that exist. In turn, the results of such studies may help to create innovative cancer-targeting therapies. Except for HNSCC, as well as cervical, ovarian, prostate, and pancreatic cancers, the Vermeer team also detected nerve markers in breast, lung, liver, and colon tumors, and found that all of these evidenced neuritogenesis in the TME, thus suggesting neuritogenesis as a general pathology among cancers. It is important to point out that for all three of the Vermeer studies cited in this article, the researchers did not systematically screen potentially cargo-carrying molecules for EVs. Accordingly, this, too, is a subject that requires additional inquiry, particularly as its results could pave the way for targeted cancer therapies.

Finally, all of the cited literature only involves a single pathogenesis—cancerous EVs inducing neuritogenesis. Additional efforts will need to focus on the role of EVs in other cancer–nerve crosstalk processes (especially on PNI and neoneurogenesis), as well as the role of EVs secreted from different cellular sources.

### 3.4. Artificial Nanovesicles Containing Peripheral Nerve Blockers Can Inhibit Cancer Progression by Targeting Cancer–Nerve Crosstalk

Given the fact that EVs play a central role in cancer–nerve crosstalk, as opposed to targeting neurotransmitter receptors in cancer cells, there may be therapeutic promise in attempting—as researchers have recently carried out in a leading-edge study—to utilize or interfere with EV-associated communication mechanisms [99]. As communication between sympathetic nerves and breast cancer has been found to exist, and genetic manipulation of autonomic innervation can inhibit cancer progression [100], these researchers have designed liposomes loaded with bupivacaine (a non-opioid, selective sodium channel blocker that interrupts the transmission of nerve impulses and pain signals), as a means to target nerves within the breast cancer TME, and thereby suppress cancer–nerve crosstalk successfully by blocking neuritogenesis and destroying neural and neuronal network integrity in the TME. Intravenously administered bupivacaine-loaded liposomes suppressed neurons in triple-negative breast cancer tumors in vivo by inhibiting carcinogenesis, invasion, and metastasis. However, although the researchers used artificial, drug-carrying vesicles to target cancer–nerve crosstalk, which objectively exploited the EV-predominant nature of nerves and tumors, they never mentioned this in their article. This is due to the fact that liposomes and EVs are such closely related bilipid-layered particles—which share a number of similarities (e.g., biodistribution, clearance rates, pharmacokinetic profiles)—and some studies have found that there is a direct competition for uptake by recipient cells between liposomes and EVs [101,102]. Additionally, both exosomes and 100 nm liposomes are readily taken up by nerves [88,99,103].

## 4. The Role of Extracellular Vesicles in Peripheral Nerve Physiology, Inflammation, Injury, and Regeneration, and How They May Be Hijacked by Cancer–Nerve Crosstalk

**Figure 3:**
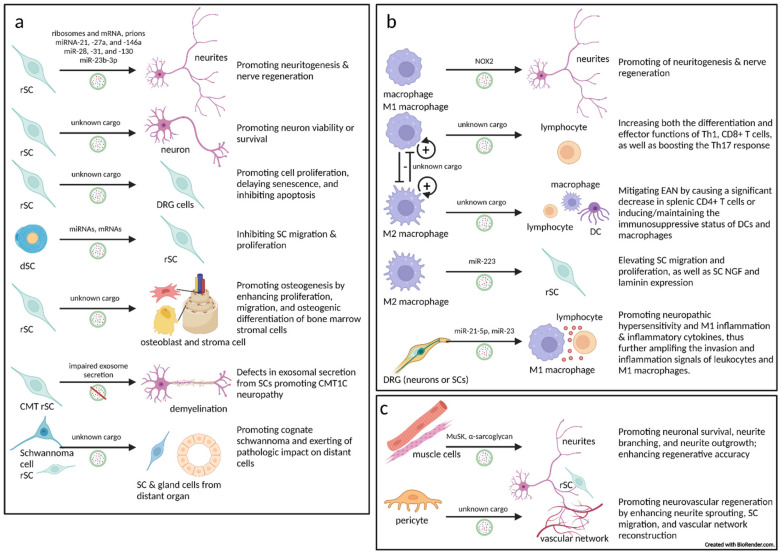
The role and mechanisms of EVs in peripheral nerve physiology, inflammation, injury, and regeneration: (**a**) Schwann cell-derived EVs (SC-EVs) have been reported to promote neuritogenesis/neuroregeneration and neuronal survival/viability; exosomal secretion defects by SCs have been shown to involve the pathogenesis of inherited demyelinating neuropathy; SC-EVs also regulate SC proliferation, viability, and migration in an autocrine manner; SC-EVs can regulate target/innervated cell proliferation and differentiation; and SC-EV functionality is largely dependent on SC phenotypes—repair (rSC) or differentiated (dSC); (**b**) Dependent upon different phenotypes (M1/M2), macrophage-associated EVs have been reported to promote neuritogenesis and neuroregeneration, to modulate the immunomicroenviornment, and to support SC functionality. Macrophages can also be regulated by neuron- or SC-derived EVs; (**c**) Innervated (target) tissue/cell-derived EVs can participate in intercellular crosstalk with neurons, axons, and SCs. In the case of cancer, under innervation, the tumor (target tissue) is involved in intercellular crosstalk with neurons, axons, and/or SCs. rSC: repair Schwann cell; dSC: differentiated Schwann cell; CMT: Charcot-Marie-Tooth (disease); EAN: experimental autoimmune neuritis (mouse model for Guillain-Barré syndrome); NOX2: NADPH oxidase 2 complexes; DC: dentritic cell; DRG: dorsal root ganglion.

Several nervous system roles played by EVs have been found to impact, both in the PNS and the CNS, neural development, physiology, inflammation, injury and regeneration, tumorigenesis, autoimmune and neurodegenerative diseases, and other typical and atypical neurological processes. Given the fact that it is unlikely that the neuronal soma could ensure an entire neuron’s constant supply solely via axoplasmic transportation [104], researchers have increasingly proven that axons and dendrites locally synthesize proteins. In recent decades, another approach, which provides these macromolecules from glial cells to axons via EVs, has been found [87,105] (Figure 3). However, to date, although information regarding the role played by EVs in the PNS is relatively scarce, it is sufficient to establish that EV communication in the PNS microenvironment is a common form of cell–cell communication.

### 4.1. The Critical Role Played by Schwann Cell-Derived Extracellular Vesicles in the Peripheral Nervous System

As the principal glia cells in the PNS, SCs regulate a wide variety of axonal functions in neural development, physiology, and pathology. Moreover, because PNS nerves can be damaged easily, SCs play a highly significant role in nerve repair and regeneration, particularly through EVs (Figure 3a).

#### 4.1.1. Schwann Cells Transfer Extracellular Vesicles to Axons

The transfer of EVs from SCs to axons was discovered in 1992 [105]. In 2004, SC cell-line-secreted EVs containing pathological prions were found [106]. In 2008 and 2011, Court et al. illustrated how ribosomes (polyribosomes and mRNA) are transferred by EVs from SCs and internalized in axons by endocytosis during axonal regeneration in rodent models [107,108]. This recovery-supportive process can largely provide damaged nerves with the ability to synthesize proteins locally.

In 2013, Court’s team further showed that rSCs (possessing strong nerve repair functions) secrete EVs, which are specifically endocytosed by axons in vitro and in vivo [88]. SC-derived EVs significantly increased axonal regeneration in vitro and enhanced regeneration after sciatic nerve injury in vivo. To confirm that the effect of axonal regeneration is dependent upon EV cargos and related to SC phenotypes, they pharmacologically induced, in vitro, these phenotypes—differentiated SCs (dSCs) and dedifferentiated, also known as rSCs—and found that EVs derived from rSCs, but not from dSCs, promote DRG explant neuritogenesis [109]. It is noteworthy that, generally, unless such inducement occurs, all in vitro SCs tend to the rSC phenotype. These observations are consistent with the role of rSCs in neural repair and tumor perineural invasion.

Through miRNA sequencing and screening, highly expressed miRNAs in rSC-derived EVs were found, several of which were associated with the regulation of neuronal processes, including axonal growth and regeneration. In this arena, the abundance of miR-21 in rSC-derived EVs is most interesting, as rSCs and their EVs have an increased expression of miR-21 compared with dSCs. The researchers further demonstrated that the proregenerative effect of miR-21 on sensory neurons comes from the amplification of the PI3K signaling pathway by targeting PTEN. [110]. In addition, the rSC-derived EV cargos of other candidate miRNAs have also been reported to be involved in diverse functions, such as cellular adhesion and immunomodulation. This is consistent with a 2019 published proteomic analysis of rSC-derived EVs, which showed that their protein cargos were also dedicated to cell adhesion, angiogenesis, and immunoresponsiveness [111]. Consequently, all of these results imply the potential roles of rSC-derived EVs to modulate angiogenesis and immunoinflammation, which may be hijacked by cancer cells to form new opportunities for cancer–nerve crosstalk.

The effects of SC-induced neuritogenesis or neural repair can be used to treat neural injury or neurodegenerative diseases. For example, diabetic peripheral neuropathy (DPN) is the most prevalent diabetes-related complication, during which comprehensive pathological changes tend to occur, including axonal degeneration and segmental demyelination, which stem from distal sensory nerves, where hyperglycemia (HG) induces SC damage and dysfunction [112,113,114]. EVs from HG-treated SCs (HG-SC EVs) can be internalized by neuron somas and axons, and locally suppress the axonal growth of DRG neurons. It was further determined that HG-SC EV-enriched miR-28, -31, and -130 potentially target DNMT3A, NUMB, GAP43, and SNAP25, thus leading to the inhibitory effect of neuritogenesis. In vivo assays produced results consistent with those derived from in vitro studies in that they showed that HG-SC EVs promote the development of DPN [112].

In contrast to HG-SC EVs, the injection of normal SC-derived EVs (SC-EVs) into the circulatory system improves the neurological function of sciatic nerves and increases intraepidermal nerve fiber density in diabetic mice without significantly ameliorating high levels of blood glucose, HbA1c, triglycerides, and body weight, thus suggesting that neural improvements may come about via the direct targeting of problematic nerves [113]. In vitro, SC-EVs brought DRG neuron neuritogenesis levels in diabetic mice back to normal and reversed HG-inhibited SC migration. Furthermore, miR-21, -27a, and -146a were found to be enriched in SC-EVs, whereby the miRNAs shuttled to and accumulated in sciatic axons, where they targeted and reduced SEMA6A, RhoA, PTEN, and pNF-κB, which are inhibitors of neuritogenesis and neural regeneration [109,113,115].

As it is known that the SC phenotype and environmental inducement can influence the EV constituents of SCs that impact neural function, stimulating SCs and altering their EV cargos through chemical or physical methods (e.g., magnetic force-based mechanical stimulation, microenergy acoustic pulse) can strengthen their neurorepair functions [116,117]. 

Interestingly, when skin precursor-derived SCs (SKP-SCs) originated from neural crest stem/progenitor cells are cultured with SC inducers, they can be differentiated to Schwann-like cells and, indeed, in nerve injury and regeneration, they have been shown to possess multiple SC functions (neuritogenic promotion, immunomodulation, etc.) [118,119,120,121]. Incorporation of SKP-SC EVs into nerves significantly facilitates neuritogenesis and myelination of regenerated axons, accelerates the recovery of motor, sensory, and electrophysiological functions, and alleviates denervation-induced atrophy of target muscles [121,122,123]. SKP-SC EVs can also be uptaken by SCs and, thereby, enhance SC viability [123]. 

#### 4.1.2. Extracellular Vesicles from Schwann Cells to Neurons

In addition to the fact that SC-derived EVs have been shown to be internalized within distal axons that promote axonal growth [112], it has been demonstrated that EVs can directly target neurons. In fact, a 2019 study demonstrated that SC-derived EVs enhanced motor neuron cell viability or survival through the inhibition of caspase-3-dependent apoptosis signaling [124]. Another recent study showed that paeoniflorin—a plant-derived monoterpene glycoside having antioxidant and anti-inflammatory biofunctions—can relieve HG damage of SC and decrease proapoptotic IRE1α pathway proteins in HG-challenged SC, thus alleviating DRG neuron apoptosis [125].

The neuritogenic and/or neuroprotective effect(s) from rSC-derived EVs to axons can be potentially employed by cancer cells to promote tumorigenesis. Moreover, rSC-derived EVs can also be incorporated directly into cancer cells and enhance disease progression, since rSC-derived EV cargos can activate several key cancer-promoting pathways.

#### 4.1.3. Extracellular Vesicles from Schwann Cells to Schwann Cells

Using RSC96 (an SC cell line)-derived EVs, it was shown showed that RSC96 EVs had a protective and reparative effect on DRG cells (mainly/probably SCs) after mechanical injury via in vitro cell proliferation promotion, senescence delay, and apoptosis inhibition by activating Wnt/β-catenin and its downstream pathways [126,127] Coculturing of SC or SC conditional medium with EVs can further strengthen the neuron-protective effects of electrical stimulation (a therapy for neural injury-related diseases), which enables DRG cell viability and inhibits apoptosis [128].

A 2019 investigation observed that dSC-derived EVs inhibited SC migration, while rSC-derived EVs did not [129]. This supports the aforementioned study in which rSC-derived EVs were shown to reverse HG-inhibited SC migration [113]. Further analysis confirmed that EVs released from different SC phenotypes can regulate their own migration and proliferation through the alteration of miRNAs and mRNAs. It is highly probable that these rSC-derived EVs and their cargos may enhance rSCs themselves and thereby to potentiate cancer progression when SCs encounter cancer cells and commence cancer–nerve crosstalk.

#### 4.1.4. Extracellular Vesicles from Schwann Cells to Innervated (Target) Tissue

The neuroprotective and regenerative role of SC-derived EVs can also help in bone regeneration since bone marrow is highly innervated, and the PNS is necessarily involved in osteogenesis, bone metabolism remodeling, and wound healing [130,131]. Specifically, rSC-derived EVs foster osteogenesis in vitro by promoting the proliferation, migration, and osteogenic differentiation of bone marrow stromal cells, and significantly enhance the therapeutic efficacy of porous titanium alloy scaffolds for bone formation and regeneration in vivo [132,133]. In the case of cancer, as a result of innervation, target tissue becomes cancerous and thus SC-derived EVs can be directly uptaken by cancer cells to promote tumorigenesis.

#### 4.1.5. Inherited Peripheral Demyelinating Disease Can Be Largely Contributed to by Extracellular Vesicle Secretion Defects in Schwann Cells

The pathogenesis of CMT1C—a rare subtype belonging to the CMT1 demyelinating family of Charcot–Marie–Tooth (CMT) disease (a common inherited neurological disorder of the PNS)—helps to underscore the critical role of SC-derived EVs [134,135,136]. CMT1C presents autosomal-dominant demyelination, which can be explained by mutations in the small integral membrane protein of the lysosome/late endosome (SIMPLE) [132,134,135,136]. SIMPLE is responsible for the formation of multivesicular bodies (MVBs) and resides within the intraluminal vesicles of those MVBs and is responsible for the release of exosomes (in this case, not involving other EV subtypes) [135]. SIMPLE mutation results in the malformation (vacuolated appearance) of MVBs, which contribute to exosomal secretion defects in multiple cell types, particularly in SCs. Defects in exosomal secretion from SCs and exosome-mediated intercellular communication help to explain the pathogenesis of CMT1C neuropathy [135]. In addition, the mutation/deletion of other vesicular trafficking proteins (including Rab7, SH3TC2, MTMR2, MTMR13/SBF2, and FIG4) account for other CMT subtypes (CMT2B, CMT4C, CMT4B1, CMT4B2, and CMT4J, respectively) [135]. These facts underscore the essential role played by SC-EVs in peripheral nerve physiology.

#### 4.1.6. Schwannoma or Schwann Cell-Derived Extracellular Vesicles May Promote Cognate Schwannoma and Exert Pathologic Impact on Distant Cells

Schwannoma is a benign tumor composed of dedifferentiated SCs [137] and is therefore not considered to fall within the realm of cancer–nerve crosstalk. Instead, it is considered to be a mass of dysregulated rSCs, whose cells present the natural characteristic of SCs (i.e., EV-predominant communication) [138,139]. An interesting case study of schwannomatosis (a rare genetic disorder that results in multiple schwannomas) displayed two significant, unusual features: (1) Stimulating prostate-specific antigen secretion from the prostate (which cannot be inhibited by dutasteride) and (2) arousing a distant cognate schwannoma tumor after the resection of the initial large schwannoma [140]. The authors suggested that EV-mediated cell communication may be responsible for this occurrence because the schwannoma cells and/or adjacent dysregulated SCs could secrete EVs through the circulatory system and be uptaken by the prostate and distant pre-schwannoma cells, resulting in prostate hyperplasia and cognate schwannoma [140]. As the prostate presents strong evidence of SC involvement in cancer–nerve crosstalk [47] and SC-derived EVs contain multiple growth-promoting cargos [111,123] that can cause prostate cells to proliferate [140], it is not surprising that SC-derived EVs can promote prostate hyperplasia and even carcinogenesis. Additionally, after a relatively long period following primary tumor resection, the distant cognate schwannoma appeared [140], which suggested that these effects may be caused by non-tumor repair/dysregulated SCs that are adjacent to and shaped by the primary schwannoma.

Similar situations can be found in vestibular schwannomas (VSs) [140,141,142]. Furthermore, it has been reported that EVs derived from the VS cells of hearing-impaired patients could damage both murine cochlear sensory cells and neurons in vitro [138].

The observations from the above referenced schwannoma studies (long-distance regulation by schwannoma EVs) imply a cancer–nerve crosstalk possibility that EVs secreted from dysfunctional rSCs may be able to mediate premetastatic niche formation, particularly as EVs have already been reported as playing a role in such formation [1,143]. Given highly active, EV-mediated communication from SCs, schwannoma can be inhibited by employing genetically modified EVs carrying suicide mRNA/protein or EVs carrying caspase-1 from genetically modified schwannoma cells [132,144,145].

### 4.2. Macrophage-Associated Extracellular Vesicles in the Peripheral Nervous System

Apart from SCs, macrophages are the second most important players in the peripheral nerve microenvironment [46]. In nerve injury and regeneration, macrophages can participate in the reparative process via resident macrophages in nerve bundle layers, as well as bone-marrow-derived, migratory macrophages that travel through the circulatory system to nerve niches [41,46,57]. Similar to SCs, macrophages are highly plastic cells that can present both M1 and M2 phenotypes, and perform different functions according to microenvironmental signals [41,57]. Macrophages secrete an abundance of EVs under physiological and disease conditions, and the function of these EVs are, in turn, dependent on the macrophage phenotype in question [146,147] (Figure 3b). Within nerves, the phenotype-dependent macrophage EV functions are not only consistent with the direct role played by macrophage–cancer crosstalk in the TME, but it implies that macrophage EVs can also enhance cancer–nerve crosstalk.

#### 4.2.1. Extracellular Vesicles from Macrophages to Nerves (Axons/Neurons, and Peri/Intraneural Immunocytes)

For neuritogenesis and repair of sensory nerves to occur, injured sciatic nerves and DRGs must have an adequate supply of reactive oxygen species (ROS) [148], which is dependent, post-neural injury, on CX3CR1-dependent recruitment and activation of circulatory macrophages. Macrophages then transfer complete functional NADPH oxidase 2 (NOX2) complexes via EVs into injured axons to regulate axonal regeneration via the production of ROS, which oxidizes and inactivates PTEN, leading to the stimulation of the PI3K-AKT pathway and neural regeneration [148]. As SC EV-delivered miR-21 inhibits PTEN and amplifies PI3K in sensory nerves [109], it suggests that SCs and macrophages cooperate in nerve repair by utilizing similar EV transfer tools and targeting pathways.

In some autoimmune neuritis diseases, such as Guillain-Barré syndrome, macrophages predominately exhibit M1 status and amplify the Th1 response, thus promoting disease progression [149,150]. On the contrary, it has been shown that by inducing the M2 phenotype and Th2 response, neuritis can be terminated and nerve repair promoted within experimental autoimmune neuritis (EAN) models [149,151]. M1 macrophage-derived EVs aggravated EAN severity by increasing both the differentiation and effector functions of IFN-γ producing Th1 and CD8+ T cells (as well as boosting the Th17 response). Conversely, M2 macrophage-derived EVs exhibited the potential to mitigate EAN by causing a significant decrease in splenic CD4+ T cells or inducing/maintaining the immunosuppressive status of DCs and macrophages. In addition, there was a dose–effect relationship between concentrations of M1 macrophage-derived EVs and IFN-γ expression in CD4+ T cells. M1 and M2 macrophage-derived EVs also modulated humoral immunity, possibly via the upregulation of ICOS on CD4+ T cells and increase in germinal center reactions [149].

Macrophage-derived EVs can also regulate macrophages themselves in an autocrine-like style, thereby amplifying their influence on the neural immune environment. It has been observed that even though reported in a spinal cord injury model, M2 macrophage-derived EVs increase the percentage of M2 macrophages, while decreasing M1 macrophages, but M1 macrophage-derived EVs acted in an opposite manner. As well, M2 macrophage-derived EV-inducing phenotypic polarization might be mediated by miR-23a-3p targeting PTEN and enhancing PI3K/AKT pathways [152].

#### 4.2.2. Extracellular Vesicles from Macrophages to Schwann Cells

As SCs and macrophages cooperate in nerve repair, macrophages secret EVs, which promote rSC functionality. Preliminary research by Zhan et al., using phenotype-induced THP-1 monocyte and RSC96 SC cell lines showed that, as opposed to M1/M0 EVs, M2-like macrophage EVs elevated SC migration and proliferation, as well as SC NGF and laminin expression. In a rat sciatic nerve injury model, M2 macrophage-derived EVs significantly increased SC infiltration and axon numbers in the regeneration site [153].

#### 4.2.3. Extracellular Vesicles from Dorsal Root Ganglia Cells (Neurons or Schwann Cells) to Macrophages

DRG cells can secrete EVs, which can make contact with macrophages. In response to chemokines produced by SCs and satellite cells after PNS axon injury, inflammatory cells, especially macrophages, infiltrate injury sites and DRGs, and release cyto/chemokines, which activate vascular endothelium and alter the sensory transduction properties of nociceptive neuron somas and axons, leading to peripheral neuronal sensitization [154,155,156]. Simeoli et al. found that, following peripheral nerve injury or capsaicin activation of TRPV1 receptors, DRG sensory nerves upregulate miR-21, which accumulates in their EVs. These actions stimulate neuron somas to release more EVs that are engulfed by nearby macrophages [156]. When these EVs deliver to macrophages’ injury-induced, neuron-upregulated miR-21-5p, the macrophages are polarized toward the M1 phenotype (pro-inflammatory/pro-nociceptive) and induce inflammatory cytokines TNF-α and IL-6. This process further amplifies the invasion and inflammation signals of leukocytes, especially M1 macrophages [156]. As we have made clear that SC-derived EVs transfer miR-21 to axons and promote neuritogenesis and regeneration in injured nerves [109] and DPN [113], we also understand that miR-21-packed OSCC EVs are transferred to sensory nerves and thereby foster neural reprogramming and transdifferentiation to adrenergic nerves that reward carcinogenesis [23]. Therefore, it is possible that miR-21 is one of the most common and important nerve messengers in sensory nerve microenvironments. Another study related to the promotion of sensory neuron-derived EVs on macrophage phenotypes/inflammation produced similar findings to those of the Simeoli team. Nevertheless, this research did not well present its DRG cell identity characterization. Based on their morphology, the DRG cells appear to be more akin to SCs [157]. In context with SC-macrophage cooperation, just as macrophages can communicate with SCs via EVs, it is not surprising that SCs can also release EVs to macrophages in a reverse process. Regardless of whether the source of EVs is from neurons or SCs, it is meaningful to know that cellular crosstalk through EVs in nerve microenvironments is not uncommon.

The intrathecal delivery of nanoparticles containing miR-21 or miR-23a antagomir in a tissue-specific manner that targets neuron–macrophage crosstalk exhibits good performance to ameliorate neuropathic hypersensitivity and reduce M1 inflammation [156]. This approach can also be translated to cancer treatment by causing cancer–nerve crosstalk interference. In addition, there is evidence to indicate that the formation and exosomal release of MVBs remains exclusive to sensory neuron somas instead of axons [158]. Thus, it is worth elucidating the differences involved in cancer–nerve crosstalk between cancer-ganglion neuron bodies and cancer-axons in the TME.

### 4.3. Extracellular Vesicles from Innervated (Target) Tissues to Nerves (Neurons, Axons, or Schwann Cells)

Following a nerve injury that occurs in the PNS, it is common to maintain neuronal viability and robust axon regeneration from the parent nerve. However, the achievement of complete functional recovery depends on the appropriate and accurate guidance of regenerating axons projecting to their terminal nerve branch and organ targets [44,159].

#### 4.3.1. Extracellular Vesicles from Muscles to Motor Neurons

EVs extracted from a muscle cell line (C2C12) can be efficiently internalized by a motor neuron cell line (NSC-34) and promote neuronal survival, neurite branching, and neurite outgrowth in a dose-dependent manner [160]. In 2019, it was found that EVs derived from denervated muscles (MD-EVs), rather than naïve muscles or other non-specific EVs, possessed a significant capability to enhance regenerative accuracy (Figure 3c). This study demonstrated the critical role that neural target cell-derived EVs could play to shape anatomical regenerative accuracy, thus promoting objective-specific neuritogenesis [159]. This should inspire us to pay more attention to the specificity of innervation to cancer, as well as related signals or markers when studying cancer–nerve crosstalk, since cancer and its tissue origin is innervated (target) tissue.

#### 4.3.2. Extracellular Vesicles from Pericytes to Nerves

Blood vessels are innervated and are an essential component of target tissues and nerves, the latter of which they are dependent upon for their development and homeostasis. Pericytes—a group of heterogeneous cells, which maintain the neurovascular unit, support angiogenesis and vascular homeostasis, modulate local immune responses, and so forth—are believed to possess progenitor properties and may play a potential role in neurovascular regeneration [161,162,163]. Pericyte-derived EVs have been found to improve microcirculation and maintain the blood–nerve barrier [163,164]. In a cavernous nerve injury (CNI) model, researchers used pericyte-derived EV-mimetic nanovesicles (PC-NVs), which showed similar characteristics to natural pericyte EVs [165,166]. The results suggested that PC-NVs might be beneficial for neurovascular regeneration by promoting neurite sprouting, SC migration, and vascular network reconstruction (Figure 3c). Intracavernous administration of PC-NVs significantly increased cell survival signaling and the expression of neurotrophic factors (BDNF, NT-3, and NGF), and improved nNOS and neurofilament-positive axon density at the CNI sites, resulting in an enhancement of the erectile function of CNI mice [166].

Taken together, innervated (target) tissue cells further promote innervation, as well as neuritogenesis via the delivery of EVs to nerves or SCs, which is consistent with published discoveries that cancer cells—the innervated (target) cells—actively contact nerves and induce neuritogenesis via EVs [20,21,22,23]. 

## 5. Conclusions

Cancer–nerve crosstalk via multiple means of pathogenesis, which mimics neurobiological processes and can be neurobiologically mapped in all aspects, is a now a critical component in the ever-growing field of oncology and suggests great advances may well be gained through close, collaborative studies between cancer neuroscience researchers and neurologists. Intercellular crosstalk through EVs, by which important supplementary or regulatory cargos shuttle between cells, is crucial and common in both physiological and pathological situations. In particular, EV communication is extremely common and significant in cancer and nerve microenvironments because the three critical characteristics they share are: (1) a demand for high amounts of material and energy, (2) a high dependence on environmental signals, and (3) the requirement that their vulnerable EV cargos be protected in harsh microenvironments or transported across long distances. Therefore, cancer–nerve crosstalk as it relates to EV-based intercellular interplay is extraordinarily worthy of greater consideration and study.

As illustrated in this review, cancer researchers have already revealed several vital roles being played by EVs in cancer–nerve crosstalk in the PNS. Meanwhile, neurobiologists have discovered additional EV cellular and molecular mechanisms at work in nerve development, physiology, inflammation, injury, and regeneration.

However, there remains much to be discovered because, as of this writing, investigations have been limited to the fact that cancer EVs induce neuritogenesis, which can be mapped to the neuritogenic and neural guidance effects on nerves of EVs derived from innervated tissues or SCs. Further efforts to elucidate the roles of EVs should endeavor to more specifically:

- Define the pathogenesis of cancer–nerve crosstalk (e.g., PNI, neoneurogenesis) in primary and metastatic tumor sites.
- Consider the distinctiveness of cancer–nerve crosstalk when mapping potential mechanisms to those of neural physiology and pathology.
- Unearth other molecular EV cargo types (e.g., lncRNAs, circRNAs, metabolites, etc.) since current research is mainly concentrating on miRNAs and proteins.
- Investigate if tumor cells which metastasize to organs other than those of the nervous system can also perform cancer–nerve interactions with nerves inside of these organs (particularly by the means of EVs).
- Examine the multiple intercellular communication components that exist within neural microenvironments. In peripheral tumors, this should center on SCs, macrophages, fibroblasts, endothelial cells, mast cells, and other immune or stroma cells in nerves or the perineural niche.

With our current understanding of SC-derived EV studies, we can conclude that SC-involved, EV-mediated cellular crosstalk between multiple cells in nerves plays a critical role in PNS physiology and pathology. Whether microenvironmental, genetic, chemical, or physical, a variety of stimulatory effects/influences on SCs can result in phenotypical and functional SC alterations, which further modify the release of SC-derived EV cargos and contribute to protective, regenerative, or damage responses. SC-derived EVs not only perform functions within the local nerve niche, but can also induce distant organic and cellular impacts.

Given the fact that SC EVs and their cargos possess multiple abilities (neuritogenic induction, survival/viability promotion, apoptosis inhibition, and so forth), it is conceivable that the recruitment and hijack of SCs to the TME is an intentional act by tumor cells. This is because SC-derived EVs may (1) directly be uptaken by tumor cells and thereby promote proliferation, resist apoptosis, and facilitate PNI; (2) induce neuritogenesis/neoneurogenesis and protect neurons/axons, thereby further enhancing tumor innervation; (3) modulate immunoinflammatory responses in the TME and potentiate tumor progression; and (4) modulate the premetastatic niche and establish favorable microenvironments to support metastatic cancer cell survival and growth.

Our present, accumulated knowledge and assumptions strongly suggest the potential diagnostic and therapeutic efficacy of targeting SC EVs. Additionally, it is likely that new investigations of other cells, which are also active in EV biology and important within the neural environment, will make further discoveries regarding their participatory involvement in cancer–nerve crosstalk via EV cargo transfers.

These explorations will not only promote a comprehensive understanding of cancer–nerve crosstalk, they will drive the development of onconeurology, cancer biology, and neurobiology. As well, by targeting EVs in cancer–nerve crosstalk, it will also improve precision clinical practice as it relates to the advancement of strategic diagnostic and therapeutic innovations.

## Data Availability

Not applicable.
